# A Retrospective Cohort Study to Assess Patient and Physician Reported Outcome Measures After Decompressive Hemicraniectomy for Malignant Middle Cerebral Artery Stroke

**DOI:** 10.7759/cureus.1237

**Published:** 2017-05-10

**Authors:** Sanjay Budhdeo, Angelos G Kolias, David J Clark, Aswin Chari, Peter J Hutchinson, Elizabeth A Warburton

**Affiliations:** 1 Clinical Neurosciences, Addenbrooke's Hospital & University of Cambridge; 2 Division of Brain Sciences, Faculty of Medicine, Imperial College London

**Keywords:** decompressive hemicraniectomy, cerebral infarction, stroke rehabilitation, patient reported outcomes

## Abstract

**Introduction:**

Decompressive hemicraniectomy for malignant middle cerebral artery (MCA) infarction is known to reduce mortality. However, there are on-going concerns in terms of the quality of life in survivors. We aimed to examine the correlation between patient and physician reported outcome measures in decompressive hemicraniectomy.

**Patients and methods:**

We analyzed outcomes in 21 patients who underwent decompressive hemicraniectomy for malignant MCA infarction between September 2003 and August 2013 within a regional health system. Patient and physician reported outcome measures were collected at follow-up. These were Stroke Impact Scale (SIS) Version 3, modified Rankin Scale (mRS), National Hospital Seizure Severity Scale, Headache Impact Test and Patient Health Questionnaire for depression.

**Results:**

There was a good correlation between physician and patient reported outcome measures. The Spearman's rank correlation coefficient between mRS and structured SIS Version 3 was -0.887 (p < 0.001); with unstructured SIS results, the correlation coefficient was -0.663 (p = 0.001). There was no statistically significant correlation between life worth and modified Rankin Scale: r = -0.3383 (p = 0.087).

**Discussion:**

Our findings of a statistically significant correlation between mRS and SIS have not previously been reported in patients with this condition. These findings provide further information to inform patient and next of kin discussions regarding outcomes from decompressive hemicraniectomy in malignant MCA infarction.

## Introduction

Malignant middle cerebral artery (MCA) infarction is a result of large MCA territory stroke causing space-occupying cerebral oedema. It carries a mortality rate of 71% if left untreated [1] and usually presents with worsening clinical symptoms and conscious level within two to five days of stroke onset. Decompressive hemicraniectomy is a surgical intervention to counter raised intracranial pressure and reduce the risk of brain herniation.

Evidence for improvements in mortality and morbidity from decompressive hemicraniectomy versus medical treatment comes from six randomised controlled trials, where decompressive hemicraniectomy has been shown to decrease mortality at the expense of significant morbidity in survivors [2]. Systematic reviews have examined the quality of life after decompressive hemicraniectomy for malignant MCA infarction [3-4]. These reviews show wide variability in patient satisfaction and quality of life after decompressive hemicraniectomy; however, most do not provide details on the correlation between physician-reported measures and patient-reported outcome measures. Physician-reported outcome measures may ignore important aspects of outcomes that matter to patients which patient-reported measures are more likely to include [5-6]. Kelly, et al. have recently published results from a small cohort, where there is a poor correlation between patient- and physician-reported outcome measures. In one of the systematic reviews, the authors commented that they were unable to do so due to lack of access to individual data [3].

The current study follows up a cohort of patients who have had decompressive craniectomies for malignant MCA infarction within a regional health system in England. We wished to analyse the correlation between patient- and physician-reported outcome measures following decompressive hemicraniectomy after malignant MCA infarcts. Specifically, we examined how well the patient’s view of whether their life was worth living correlated with validated physician and patient-reported outcomes scales. In addition, we analysed how well validated patient reported scales correlated with validated clinician reported scales. In addition, we wished to examine the burden of other symptoms causing morbidity in this cohort. This included depression, headache, and seizures, known causes of reduced quality of life in this cohort [7-10].

## Materials and methods

### Patient selection

Patient selection for decompressive hemicraniectomy across the East of England was made using a stroke network protocol drawn up according to national guidelines [11-12]. Patients from eight district hospitals were transferred to the regional neurosciences centre for consideration of hemicraniectomy if they fulfilled the national criteria [12]. If patients were seen locally rather than being transferred, an additional criterion was computed tomography (CT) perfusion scanning. Patients with a large penumbra, as demonstrated by prolonged mean transit time (MTT), moderately reduced cerebral blood flow (CBF) with no derangement in cerebral blood volume (CBV), were considered for decompressive hemicraniectomy after discussion between the neuroradiology, stroke, and neurosurgical teams. Surgery was performed within 48 hours of onset of symptoms, or in a delayed fashion after symptomatic deterioration from the initial stroke insult.

### Patient follow-up

Patients were followed up as part of a service evaluation at Addenbrooke’s Hospital, Cambridge, UK. Consequently, in accordance with local guidelines, ethical approval was not sought. The patient cohort was selected by analysing records between September 2003 and August 2013. Records were collected from (i) notes kept by the stroke department specialist nurses of patients who underwent decompressive hemicraniectomy and (ii) an analysis of operation notes and operative records kept by the neurosurgery department. Out of 30, 26 were still alive at the time of contact and 21 were assessed. Five patients were lost to follow-up. Baseline demographic data is shown in Table 1. Mean follow-up duration was 36.5 months after stroke (range 6-122 months). Sixteen patients underwent cranioplasty. No patients required shunt insertion.

**Table 1:** Baseline demographic data and characteristics for the 21 patients included in this study The cohort has a balance in terms of sex and laterality of infarct. TIA: Transient ischaemic attack.

Number of Patients	21
Age (Mean ± SD)	45.6 ± 10.9
Sex	
*Male*	10
*Female*	11
Medical History	
*Smoking*	6
*Hypertension*	7
*Hypercholesterolaemia*	3
*Diabetes*	4
*Stroke/TIA*	3
*Atrial Fibrillation*	1
Pre-operative GCS (Mean ± SD)	11.7 ± 2.4
Infarct Lateralisation	
*Right*	10
*Left*	11
Time from infarct to surgery in days (Mean ± SD)	2 ± 1.7
Time to follow-up in months (Mean ± SD)	36.5 ± 27.5

Thirteen patients were seen in person. Eight of the 21 patients assessed remotely by telephone, with the help of their relatives, due to travel constraints. A number of scales were used to quantify patient experience after decompressive hemicraniectomy, with instructions to consider the period prior to cranioplasty if relevant: the Stroke Impact Scale (SIS) version 3.0 (copyright University of Kansas Medical Center) to monitor quality of life; the Headache Impact Test-6 survey to quantify severity of headache symptoms [13]; PHQ9 depression scale to quantify mood symptoms [14]; the National Hospital Seizure Severity Scale to quantify severity of seizures [15]; the modified Rankin Scale (mRS) to quantify motor function and ability to carry out activities of daily living. Where the patient had more than one type of headache or seizure, the highest scoring headache or seizure type was used for the purposes of the scales. In cases where the patient had difficulty in answering questions due to dysphasia, the assessment was undertaken with the help of patient relatives. Patients also answered a customised questionnaire for the service evaluation, to determine the quality of follow-up services, which are not reported in this paper.

### Data analysis

**Figure 1:**
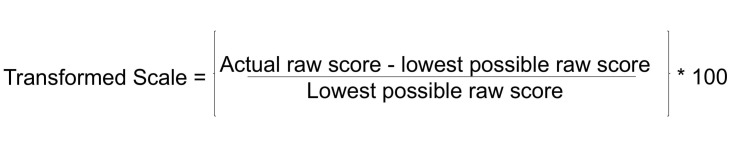
Equation for transformation of Stroke Impact Scale values to standardised form

Analysis of data was carried out using SPSS Statistics for Windows, Version 23 (IBM Corp, Armonk, NY) and Microsoft Excel 2010. For the stroke impact scale, raw scores were transformed to standardised scores using the formula shown in Figure 1.

## Results

**Figure 2:**
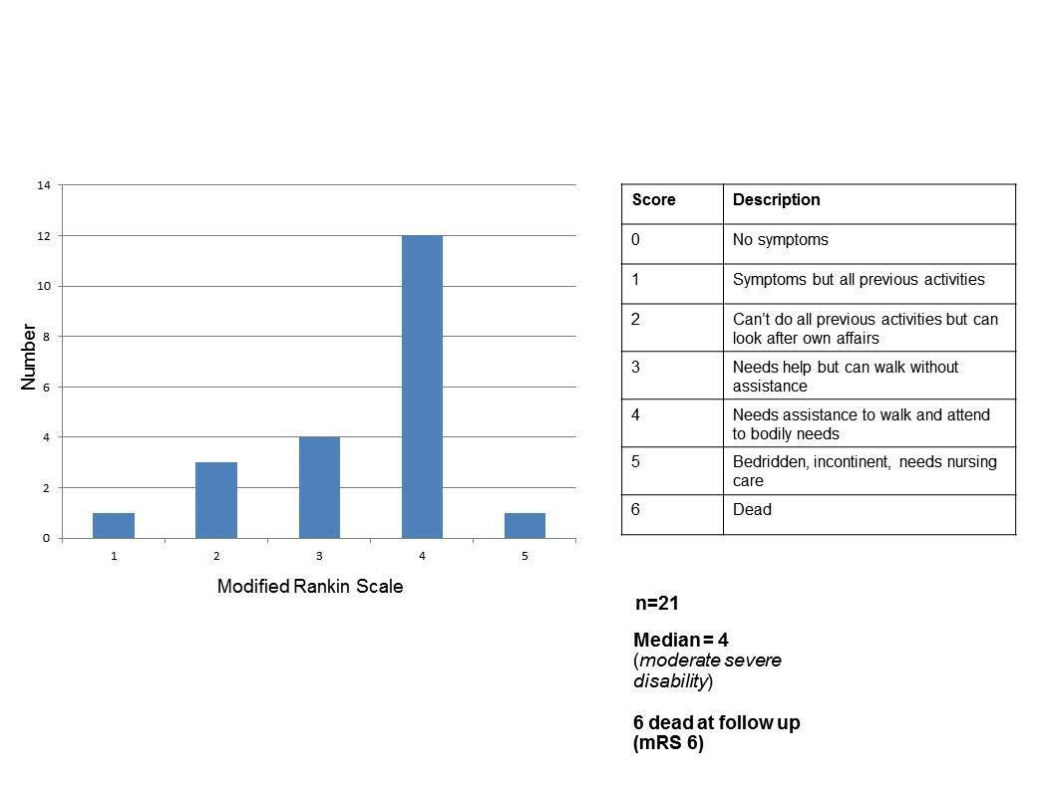
Modified Rankin Scale results for the cohort The results confirm distributions of scores which have been found in previous study, which suggest that the modal mRS score at follow-up is 4. This study has longer follow-up period than other studies and therefore confirms this is the case over a longer period. Due in part to length of follow-up, there is a number of patients in the cohort who were dead at the time of recruitment. mRS: modified Rankin Scale.

Mean age at stroke onset within this group was 45.0 (age range 18-63 years). A bar chart of the modified Rankin scale results is displayed in Figure 2. There is a reasonable spread of results, with a standard deviation of 0.98. Of the 21 patients studied, four patients had a modified Rankin scale of 2 or better, conventionally regarded as a good functional outcome [16]. In addition to the patients seen, there were four patients who would score 6 because they died prior to the follow-up period of the study.

**Figure 3:**
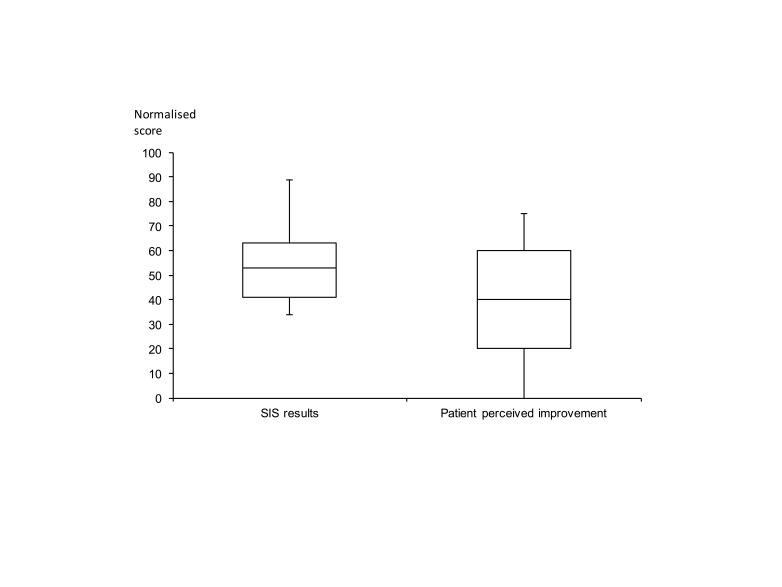
Stroke Impact Scale results There is a wide distribution of outcomes in the structured (SIS results) and unstructured (patient perceived improvement) parts of the questionnaire. For the structured questionnaire, this was true of all domains except that examining the function of the affected hand. SIS: Stroke Impact Scale.

A boxplot of the SIS results is displayed in Figure 3. The structured questionnaire was completed as well as a question asking patients to quantify their perceived extent of recovery. The standard deviation of the unstructured question (26.0) and the structured questionnaire (15.6) suggests that there is a widespread in responses to patient-reported recovery outcomes. With regard to the structured questionnaire, there is wide variability in the results for all domains, except for the function of the affected hand, where this is uniformly poor function. For each domain of the SIS, scores were as follows (mean ± standard deviation): strength 16.7 ± 18.5; hand function 2.9 ± 10.7; mobility 35.6 ± 25.8; ADL/IADL 34.2 ± 19.9; emotion 54.5 ± 18.3; memory 46.9 ± 25.5; communication 48.6 ± 25.9; participation 29.5 ± 18.7; stroke recovery 28.3 ± 26.4). There was a wide distribution in outcomes in each of the individual domains, except for the affected hand domain.

By obtaining physician- and patient-reported outcome measures, we have been able to examine how well they correlate. To determine the use of correlation coefficients, the Shapiro-Wilk test has been used to test for normality at the p = 0.05 level. Unstructured SIS results and mRS results did not meet criteria to be considered normally distributed. The structured SIS results met criteria to be considered normally distributed. Consequently, Spearman's rank correlation coefficient has been used for comparisons.

**Figure 4:**
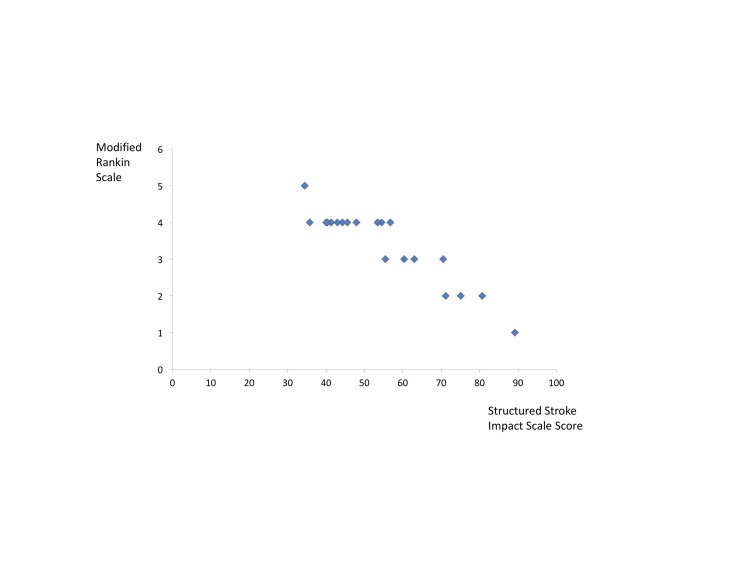
Correlation between Stroke Impact Scale and modified Rankin Scale Examining the association between modified Rankin Scale (mRS) and the structured Stroke Impact Scale (SIS) results, the Pearson correlation coefficient was 0.929 (p = 0.000). Results from the structured SIS were used. The outcome suggests strong correlation, which was highly statistically significant.

Figure 4 demonstrates the association between mRS and the structured SIS results. The Spearman's rank correlation coefficient was -0.887 (p < 0.001). Examining the association between mRS and the unstructured SIS results, the Spearman's rank correlation coefficient was -0.663 (p = 0.001). In these instances, there is a strong correlation between the patient and physician recorded outcome measure, which is statistically significant. The Spearman's rank correlation coefficient between the structured and unstructured SIS was 0.750 (p < 0.001). The correlation between one of the questions in the stroke impact scale – “Is life worth living?” – and the modified Rankin Scale was calculated. The question was rated on the score of 1 to 5, with the ratings reversed as per guidance from the SIS, such that 1 means none of the time, 3 means some of the time, and 5 means all of the time. The Spearman's rank correlation coefficient was -0.3383 (p = 0.087), indicating no statistically significant correlation between the mRS and the 5-point satisfaction with life scale of the SIS.

Results from the PHQ9 depression scale indicate that 47% of the patients did not report answers suggesting depression. Of those that were depressed, there was an even spread between mild, moderate and moderate-severe depression, with 19% having mild depression, 10% having moderate depression, and 19% having moderate-severe depression. There were no patients that were severely depressed. Patients with a favourable mRS (1 and 2; n = 4) reported no depression. Of the 17 patients with an unfavourable mRS (3-5), six reported no depression (35%), four had mild depression (24%) while seven had moderate or moderately severe depression (41%). There was a moderate correlation between PHQ9 and both physician and patient-reported outcome scales. The correlation of PHQ9 with mRS was 0.591 (p = 0.005), the correlation with structured SIS was -0.605 (p = 0.004). In contrast, there was poor correlation with the unstructured SIS (r = -0.456, p = 0.038) and with the question of whether life was worth living (r = -0.347, p = 0.123).

Nine patients out of the 21 subjects reported having headaches after decompressive hemicraniectomy. Headache severity was measured with the HIT-6 questionnaire. Of those patients suffering from headaches, mean severity was 52.6 ± 9.2 (mean ± standard deviation), out of a possible total of 78 which implies some impact on life, sufficient for medical review to be recommended. Ten patients out of the 21 subjects reported having seizures. Seizure severity was measured using the National Hospital Seizure Severity Scale. Of those patients with seizures, mean severity was 11.0 ± 6.1 out of a possible total of 27. For comparison, a nationally representative cohort of epilepsy patients had a mean score of 14.2 [17].

As part of a customised questionnaire, subjects were asked about their ability to resume their pre-morbid working lives. Seventeen patients (81%) were not working at the time of follow-up. Three patients (14%) had a restricted return to work. Either they had to work fewer hours than they previously had done, or they had started a different job that was not as physically demanding. One patient (five percent) had returned back to previous work.

## Discussion

### Principal findings

In this detailed follow-up study of 21 patients who have undergone decompressive hemicraniectomy for malignant MCA infarction at a single centre, we found wide inter-individual differences in outcome measures.

There is a strong relationship between physician-reported outcome measures and some patient-reported measures measured through validated scales. Interestingly, there is a strong correlation between structured SIS and unstructured SIS, implying that the structured questions accurately represent the quality of life domains in this cohort. Depression was poorly correlated with whether subject found life worth living.

Analysing measures of disability and life satisfaction in the British Household Panel Survey, people who become disabled undergo a decrease in mean life satisfaction, but this partially recovers with time [18]. Semi-structured interviews in stroke patients reveal that they value the meaning behind activities that they are no longer able to do [19]. This study suggested that adaptability was a second vital factor to life satisfaction, in addition to extent of deficits. Semi-structured interviews in patient groups with other disabilities resulted in similar conclusions [20]. A third factor known to determine the quality of life in other conditions is the extent of social interactions a person has [21-22]. Activities and the extent of social interactions are assessed as part of the structured SIS.

### Comparisons with previous research

The spread of mRS is broadly similar to that of previously published research [1-3]. The modal mRS score of 4 accords with a meta-analysis of HAMLET, DECIMAL, and DESTINY trials [2]. SIS version 3 results in each subdomain from our study are broadly similar to the previously published results for SIS Version 2 [1].

Kelly, et al. reported a Spearman's rank correlation coefficient of -0.44 (p = 0.2) for mRS and SIS-16, in contrast to our findings regarding mRS and SIS Version 3, which showed a strong, statistically significant negative correlation [23]. There are three possible explanations for this discrepancy. First, our patient reported outcome measures examine different domains. The Stroke Impact Scale Version 3 also features questions on memory and thinking, mood, communication, and activities that give purpose in life, which the SIS-16 does not focus on. While the inclusion of factors not related to physical function might be expected to produce a poorer correlation between the two measures, the domain exploring the activities that subjects undertake may be a good indication for motivation in reaching functional recovery, and inclusion of these activities may itself act as further motivation to improve functioning [24]. Second, there are sample size differences between the two groups. Kelly, et al. only collected outcomes for 11 patients, so the study was more highly powered.

Rahme, et al. reviewed various aspects of quality of life after decompressive hemicraniectomy for stroke [3]. Their work suggested that the majority of this patient cohort thought life was worth living after stroke. In a sample of 209 subjects that they analysed, 160 subjects (76.6%) were satisfied with life. In a 5 point selection scale similar to that used in our study, Pillai, et al. obtained a score of 4.4 ± 1.2 for life satisfaction [25]. Ragoschke-Schumm, et al. recorded results from a 4 point life worth living scale (lowest value meaning highest satisfaction). They had a mean of 2.0 for age under 60 and 2.18 for 60 and over [26]. Our results were in between the values from these studies.

Rahme, et al. also systematically reviewed research on depression after decompressive hemicraniectomy for malignant MCA infarct. A variety of methods were used to determine depression, making conclusions on the severity of depression difficult. In studies where evidence regarding depression was systematically collected, 64 out of 116 subjects (55.3%) reported depression, with 52 (44.7%) reporting no depression. In our study, 10 out of 12 subjects (47.6%) did not report depression, which is consistent with previous studies. There was a moderate correlation between mRS after decompressive hemicraniectomy and depression. Our findings are consistent with results previously reported by Kelly, et al. (r = 0.3, p = 0.2) [23]. It is possible that depression had a deleterious impact on outcomes, perhaps because low mood impacts participation in rehabilitation [27], or makes it more difficult to adapt to new circumstances.

### Limitations

This study adds to the "real life data" outside of randomized control trial criteria for enrollment, which are often stricter than clinical criteria for undertaking decompressive hemicraniectomy; however, as a retrospective single centre study, this study has some limitations.

First, given the time period over which patients were eligible for recruitment, the follow-up period has varied from six months to 122 months. Kelly, et al. studied 11 patients over early (3 ± 2 months) and late (9 ± 3 months) follow-up periods [23], and found that there was a statistically significant difference between patient recorded outcomes scores between periods, so the time period may have had an effect on results. Second, in common with other previously published observational studies, this study has no control group. Third, this study is limited by the small sample size, in common with much of the literature in this condition. Specifically, for this study, the sample size was reduced from 30 to 26 due to patient death and five further patients were lost to follow-up. The small sample size is partly due to a historical low referral rate to the regional centre for neurosurgery. Referral rates have now increased and the operation is carried out more commonly. Fourth, detailed psychiatric assessment for this cohort was not possible; a validated scale for mood (PHQ9) was used instead. This carries the advantage of an ability of looking at quantifiable differences in scores. A specific disadvantage of PHQ9 is that it has good sensitivity but a poorer specificity for depression [28]. Fifth, the study was carried out in the same unit where patients had their operations. However, this is true of most of the previously published observational studies in this area [23-25, 27], and assessors had not previously been involved in the clinical care of the patients concerned.

### Future research

Research to date examining the quality of life after decompressive hemicraniectomy for malignant MCA infarcts has followed small cohorts. A coordinated effort using a common set of questionnaires would be useful to confirm our findings. The effect of pre-morbid psychologic state and resilience on depression and quality of life after stroke is unknown. Studies, which are able to collect evidence regarding subjects’ premorbid state, would be particularly useful in examining whether this information would have prognostic value. Were this to be the case, it would be valuable for family discussions, decisions regarding surgery and prognostication. Existing studies have generally had one or two follow-up points. Multiple follow-up points would be useful to establish whether there are changes in physician and patient reported outcomes with time.

## Conclusions

We report that in patients undergoing decompressive hemicraniectomy for malignant MCA infarction, there are statistically significant negative correlations between mRS and SIS Version 3, as well as between mRS and the unstructured SIS reports. The novel nature of these findings can be explained in two ways. First, the structured SIS Version 3 contains domains which examine involvement in activities which may be a causal or consequential measure in functional recovery, or both. Second, our study has a larger sample size than a previous study examining the correlation between patient and physician outcome measures which did not find a statistically significant correlation.

These findings provide further information to inform patient and next of kin discussions regarding outcomes. There are two important points to make in such discussions, arising from this study. First, this study demonstrates the importance of talking about the patient’s view of an outcome and how it might differ from objective outcome measures. Second, this study shows the complexity of determining what constitutes a good outcome – mood may not necessarily be correlated with functional recovery.
